# “I'm Pregnant, What Do I Do?”: Exploring How People Having Abortions in Britain Find and Use Online Sources of Information

**DOI:** 10.1111/psrh.70016

**Published:** 2025-06-27

**Authors:** Rosa Mackay, Rachel Scott, Maria Lewandowska, Rebecca Meiksin, Natasha Salaria, Patricia A. Lohr, Sharon Cameron, Melissa Palmer, Rebecca S. French, Kaye Wellings, the SACHA Study Team, Annette Aronsson, Paula Baraitser, Caroline Free, Louise Keogh, Clare Murphy, Wendy Norman, Jennifer Reiter, Jill Shawe, Sally Sheldon, Geoffrey Wong

**Affiliations:** ^1^ Department of Primary Care Health Sciences University of Oxford Oxford UK; ^2^ Faculty of Epidemiology and Population Health London School of Hygiene & Tropical Medicine London UK; ^3^ Faculty of Public Health and Policy London School of Hygiene & Tropical Medicine London UK; ^4^ Centre for Reproductive Research & Communication, British Pregnancy Advisory Service Leicester UK; ^5^ Queen's Medical Research Institute, University of Edinburgh MRC Centre for Reproductive Health Edinburgh UK

## Abstract

**Background:**

Accurate, timely, and supportive information is important for high‐quality abortion care. Limited research explores how people find and use online sources of information (OSI) during the abortion process, particularly in Britain. Understanding experiences of using OSI is important for the development of person‐centered services and resources.

**Methods:**

We conducted a thematic analysis of qualitative data from 41 semi‐structured interviews carried out in 2021/2022 with people who had recent experience of abortion in Britain.

**Results:**

Using OSI was common amongst participants. Our analysis generated four distinct motivations for doing so. People used OSI to find information about accessing abortion services. OSI was also used to demystify abortion, as many participants did not understand what the process involved or know what to expect. Connection and solidarity were sought through OSI, and some participants felt supported by the content they found, particularly through the accounts of others. Finally, people used OSI to explore their relationship with their pregnancy during the abortion process. Using OSI brought benefits—including finding non‐judgmental and supportive resources—and challenges, including struggling to find relevant information or encountering negative stories and anti‐abortion views. Nonetheless, participants appreciated OSI and expressed a desire for more real‐life stories and online spaces where they could connect with others.

**Conclusions:**

People having abortions want and need different things from the online resources they consult. However, OSI have the potential to provide valuable information, connection, and a place for exploration around the topic of abortion. Future work should explore how OSI can meet these different needs, guided by the motivations of users.

## 1 Introduction

Accurate, timely, and supportive information is important for high‐quality abortion care [1, 2]. Due to the time‐sensitive nature of abortion, resources must be quickly and easily accessed. Poor‐quality information that is inaccurate or not geographically specific, or an absence of any information at all, can be a significant barrier to care and limit the choices that are available to people regarding their pregnancy [3]. This article explores how people who recently had an abortion in Britain found and used online sources of information (OSI) throughout their abortion process. The abbreviation “OSI” is used for the purposes of ease and is not a formal acronym.

In Britain, 99% of abortions are National Health Service (NHS)‐funded, provided free of charge at specific NHS sites or by NHS‐contracted independent providers [4]. In England and Wales, the majority of abortions are carried out by these independent providers, including BPAS (British Pregnancy Advisory Service), MSI Reproductive Choices, and NUPAS (National Unplanned Pregnancy Advisory Service) [4]. In Scotland, where abortion policy varies slightly due to the devolution of power to NHS Scotland, the majority of abortions are provided directly by the NHS. Across Britain, services are accessed either through self‐referral directly with the provider or referral via a general practitioner (GP) or sexual health clinic.

The abortion rate across Britain has been increasing year on year [4, 5]. Alongside this growing demand for services, the landscape of care has changed significantly. In 2020, the Covid‐19 pandemic prompted a telemedical model to be more widely adopted, facilitating remote access to consultations and fully home‐managed medication abortion (MA), in which both sets of pills are taken outside of a clinical setting. As such, in England and Wales, there were no entirely home‐managed abortions in the first quarter of 2020, whereas these represented 52% of all MA in 2021 [4]. A recent review highlighted the need to consider the additional work carried out by people to prepare for and have an abortion at home, practically and emotionally, and to understand the information and assistance required to ensure they feel confident and supported throughout their abortion [1]. A need for greater emotional and interpersonal support through the abortion process was highlighted in work that reported on the experiences of people seeking abortions in this current landscape of provision [6].

Although abortion access is largely supported by the British public [7] and the procedure is common, approximately one in three women will have one in their lifetime, [8] stigma remains highly relevant to abortion experience and an important issue for access to and quality of care [9]. In 2009, Kumar, Hessini, and Mitchell defined abortion stigma as a “negative attribute ascribed to women who seek to terminate a pregnancy that marks, internally or externally, as inferior to ideals of womanhood” [10]. Abortion stigma is informed by sociocultural, political, and ideological factors, in turn, influencing the narratives and representations of abortion within institutions, public discourse, popular media, and online spaces [11]. Stigma, both anticipated and experienced, can limit abortion disclosure and create barriers to health services, impeding people's ability to obtain information, seek care, and receive support [12].

OSI have long been established as an important resource for those with stigmatized health conditions or experiences, including abortion [13, 14]. Online spaces can provide not only practical information but also a space for sharing personal experiences and accessing social resources where negative attitudes and stigma may hinder a person's ability or willingness to seek support in person [15, 16]. Recent work has suggested that people seek accounts of other people's abortion experiences online to meet an unmet need for in‐person support in their own social networks [17]. However, while the internet is able to provide a level of anonymity, which can impart a sense of protection from stigma, stigmatizing, or anti‐abortion views can still be encountered. As such, there has been growing discourse around the opportunities and challenges of finding and sharing abortion information online. This includes information on video‐sharing platforms such as TikTok [18], first‐person online storytelling mediums [11], and public awareness campaigns aimed at reducing abortion stigma such as the #ShoutYourAbortion trend on X, formerly Twitter [19].

Studies on the use of the internet to seek abortion information have largely focused on: exploring how people access abortion services [20]; evaluating OSI quality and content [21, 22]; understanding how people interpret information they find [23]; and the demand for abortion information [24, 25]. These frequently come from a US context, representing a different policy and legislative environment to that of Britain. Furthermore, research in this area frequently explores a single online platform or resource, such as Reddit [26, 27, 28, 29, 30, 31, 32], rather than exploring how people engage with a variety of sources.

Few studies have explored the experience of using OSI for abortion, considering how and why a range of OSI are used as people navigate their abortion process. As people's perception of abortion can be significantly influenced by information found online [23, 33], it is pertinent to understand current practices and motivations around OSI use. Furthermore, following a period of policy change in Britain initiated by the Covid‐19 pandemic, developing an understanding of people's experience of abortion under new models of care is critical. This article will explore how people having abortions in Britain find and use OSI by describing the types of OSI people engage with, why they seek the information they do, and their experiences of doing so. This work will contribute to literature on the use of the Internet for abortion information and has scope to inform and support abortion services in Britain by considering the needs and experiences of service users.

## 2 Methods

This article reports on data from the SACHA Study: Shaping Abortion for Change (www.lshtm.ac.uk/sacha), which was designed to create an evidence base to inform future developments in abortion care in Britain. This work was approved by LSHTM (Ref: 22761), NHS (IRAS Project ID: 291993), LSHTM MSc Research Ethics Committee (Ref: 29518), and BPAS (Ref: 2021/02/WEL).

### 2.1 Participant Selection

Between July 2021 and August 2022, we purposively sampled 48 people with recent (within the last 2–8 weeks) experience of having an abortion on the basis of age, ethnicity, and method of abortion. Recruitment was carried out at six sites across England, Scotland, and Wales, from both NHS abortion services and NHS‐funded independent services. The inclusion criteria were: aged 16 years or older, a UK resident, able to provide informed consent, and able to speak a language that was available to a member of the research team (Arabic, English, Polish, or Welsh). Furthermore, only those choosing abortion on grounds other than fetal anomaly were included. While some features overlap, abortion for fetal anomaly involves motivations, circumstances, and experiences that are beyond the scope and objectives of this study.

### 2.2 Data Collection

We carried out semi‐structured interviews between September 2021 and August 2022 by phone or video conferencing software, depending on the participant's preference. We obtained verbal consent for the interviews, which were audio‐recorded and transcribed verbatim. In recognition of their contribution, we offered participants a £20 shopping voucher. All the interviews were conducted in English, except one which was carried out in Polish. At the request of the participant, this interview was not audio recorded. Instead, detailed notes were taken in English by the researcher (M.L.) which were then used in data analysis.

The interview topic guide was designed to capture people's experience of their recent abortion. To do so, it covered all aspects of the abortion process: pregnancy identification, decision‐making, help‐seeking and referral, the abortion procedure, after‐care, the impact of Covid‐19, and views on service provision and support. There were no specific questions about the use of OSI in the topic guide; discussions around this practice arose unprompted. Thus, the topics explored in this article are grounded in the experiences of the participants. Depending on the nature of the interview, however, some participants may not have discussed OSI despite having engaged with online resources. Our data may have, therefore, not captured the full picture of OSI use.

### 2.3 Data Analysis

Seeking to explore how resources were used that could, in theory, be accessed by any person via the internet, we considered OSI as distinct from online information provided as part of an individual's formal pathway of care. Thus, data relating to email communication and video consultations with providers or medical professionals were not included in the analysis. Information from providers and healthcare organizations found through publicly accessible platforms was, however, included. For the purposes of this paper, OSI include any information in text, audio, video, or pictorial form accessed via internet platforms, including but not limited to: search engines (Google), location‐based directories (Google Maps), news media (BBC News), video sharing and entertainment platforms (YouTube, Tik Tok), audio platforms (Spotify), social networks (Facebook, Reddit), and app stores (Google Play).

We used the Framework Method, a type of thematic analysis [34, 35], to analyze the interview transcripts, drawing on Clarke and Braun's guidelines for qualitative analysis [36]. This method involved five steps: (1) familiarization; (2) building a coding index; (3) indexing; (4) charting and summarizing; and (5) interpretation.

R.Ma. and R.S., both of whom read all the transcripts in full, conducted the analysis. The lead author, R.Ma., began the analysis process by familiarizing themselves with a randomly selected subset of the interview transcripts. Upon second reading, R.Ma. open coded these transcripts by hand, allowing codes to be generated inductively, informed by relevant literature and the research aims. At this stage and all further stages of the analysis, only sections of the transcripts where participants discussed OSI were coded, and thus the final dataset included instances across the transcripts where this topic was mentioned. Of the 48 participants interviewed, 41 described having used OSI during their abortion process. Seven participants did not refer to this topic in their interview, and as such did not contribute to the dataset. In some cases, there were challenges in identifying OSI use where participant descriptions implied an online source but did not include explicit mention of OSI or searching online. In these instances, data were included only where it was clear that a participant had engaged with or expressed views on OSI.

R.Ma. and R.S reviewed the initial codes together, which informed the creation of the coding index. Codes were combined into clearly defined, higher order categories where appropriate, continuously referring to the original text to avoid losing context. Thus, the coding index consisted of a collection of themes and subthemes represented by categories and nested codes.

Using NVivo 12 software, R.Ma. systematically applied the coding index to the 41 transcripts in which OSI were mentioned, labeling the dataset with existing codes and adding to the index where new labels were needed to best describe the data. The process was highly iterative, and R.Ma. and R.S. had frequent meetings to discuss the coding process. Analytical and reflexive memos were written throughout to track ideas about and connections within the data.

Once the coding index had been applied to all the transcripts, R.Ma. and R.S. began charting the data by organizing themes into a discernible hierarchy. This was then transferred to a matrix where the dataset was summarized by case and by theme, using verbatim excerpts from the transcripts and written summaries. Aided by this matrix and analytical memos, R.Ma. and R.S. interpreted the data relative to the research aims.

## 3 Results

Participants in the study who discussed OSI (*n* = 41) were aged between 16 and 39 years. Ethnicity was self‐defined, and while there was a wide diversity of ethnicities recorded, the majority of participants were white. The sample included 23 abortions carried out in England, 16 in Scotland, and 2 in Wales. Thirty three of these were home MA, six were procedural abortions, one was a failed home MA followed by a procedural abortion, and one was a hospital MA. These characteristics are broadly representative of the wider British population, particularly regarding the method of abortion [4]. Many participants had previously had an abortion(s). Sample characteristics are further detailed in Table 1. Quotes are presented with the assignment of a participant identifier and characteristics, “[P#, age, ethnicity, abortion method]”.

**TABLE 1:** Sample characteristics of participants included in analysis (*n* = 41).

Participant ID	Age	Ethnicity	Children at time of interview	Abortion method	Previous abortions	Country of abortion
P01	31–35	White British	No	Home MA	No	England
P02	26–30	White British	No	Home MA	No	England
P03	21–25	British Bangladeshi	No	Home MA	Yes	England
P04	26–30	White British	No	Home MA	No	England
P05	36–40	White British	Yes	Home MA	Yes	England
P06	36–40	White British	Yes	Home MA	Yes	England
P07	41–45	White British	Yes	Home MA	Yes	England
P08	31–35	White British	No	Home MA	Yes	England
P09	21–25	White British	No	Home MA	No	Wales
P10	31–35	White British	No	Home MA	Yes	Scotland
P11	21–25	White British	No	Home MA	No	Scotland
P12	26–30	White Irish	No	Home MA	Yes	England
P13	21–25	White British	No	Home MA	No	England
P14	26–30	White British	Yes	Home MA	No	Scotland
P15	31–35	White British	Yes	Home MA	No	Scotland
P16	31–35	White Polish	No	Home MA	No	Scotland
P17	26–30	White British	No	Home MA	No	Scotland
P18	36–40	White Canadian	No	Home MA	No	Scotland
P19	21–25	White British	No	Home MA	No	Scotland
P20	36–40	Not specified, Hungarian	Yes	Procedural	Yes	England
P22	26–30	White British	No	Home MA	Yes	Scotland
P24	26–30	White British	No	Home MA	No	Scotland
P25	26–30	White British	Yes	Home MA	Yes	England
P26	21–25	White British	No	Procedural	No	England
P27	31–35	White British	Yes	Procedural	No	Scotland
P28	26–30	White Hungarian	No	Home MA	Yes	Scotland
P29	21–25	Pacific Islander	No	Home MA	No	Scotland
P30	31–35	White British	No	Home MA	No	England
P31	36–40	White British	No	Home MA	Yes	Scotland
P32	16–20	White British	No	Home MA	No	Scotland
P33	31–35	White British	Yes	Home MA	Yes	England
P35	31–35	White British	Yes	Home MA	No	England
P36	36–40	Black African	Yes	Procedural	No	England
P37	21–25	White British	No	Home MA	No	England
P38	16–20	White Asian	No	Home MA	No	England
P40	31–35	Asian Nepali	Yes	Procedural	Yes	England
P42	31–35	Black British	No	Procedural	No	England
P43	16–20	Asian Afghani	No	Home MA	No	England
P44	16–20	White British	No	Home MA	No	Wales
P45	21–25	White British	No	Hospital MA	No	Scotland
P48	21–25	Middle Eastern	No	Home MA (failed) + Procedural	No	England

Abbreviation: MA, medication abortion.

In this paper, both “person/people” and “woman/women” are used to describe those who are having abortions, to acknowledge that abortions are not only experienced by cisgender women, but also by intersex, non‐binary, and transgender people. As no participants in this study disclosed a gender identity other than that of a woman, these terms are used interchangeably when reporting the results. When discussing the implications of this research, care has been taken to use gender‐inclusive language. Furthermore, when reporting our results, we have chosen to adopt the language individual participants used when describing their pregnancy, be that “fetus”, or “pregnancy.”

OSI mentioned in participant accounts included: search engines such as Google; “formal” sources such as health information or provider websites from organizations like the NHS or BPAS; digital pregnancy and period tracking apps; article‐based platforms including blogs; and username‐based forums such as Reddit. Participants referred to the use of OSI throughout the abortion process, and motivations for doing so guided the way they described their experience. We categorized these motivations in four ways to build an understanding of how OSI were used: (1) accessing services, (2) demystifying abortion, (3) connection and solidarity, and (4) engaging with pregnancy content. In many cases, participants used OSI at multiple stages of their abortion process and expressed a variety of motivations.

### 3.1 Accessing Services

The use of OSI appeared early in most accounts when participants discussed initiating the process of accessing care and finding abortion services. Search engines were often the first place people went: they looked broadly for information on how to begin the abortion process, where they could go, and whom they could contact.

To be honest I did Google “I'm pregnant, what do I do” kind of thing. [P11, 21–25, white British, home MA]

Service information was usually sought from healthcare and provider websites. One woman explained that she searched online because she didn't know where to turn [P06, 36–40, white British, home MA]. Most participants described this process as “straightforward” [P04, 26–30, white British, home MA], and reflected that it was easier than they had anticipated to find the information they needed. People expressed appreciation and surprise around the quantity of information that was available and the “non‐judgemental” [P01, 31–35, white British, home MA] nature of advice on provider websites.

However, some participants also described issues that arose around obtaining the information they needed from provider websites. These included finding incorrect phone numbers and opening hours, out‐of‐date information, and limited area‐specific advice. One person reported that it took time to search for a provider, reflecting on how the lack of open discussion about abortion impacted her ability to find the information she needed.

… if it was talked about a little bit more I would maybe know that 80% of abortions are supplied by BPAS therefore instead of trying to Google, like try and find if it was like something offered by the NHS or BPAS … [P13, 21–25, white British, home MA]

In the only case in our sample where someone struggled to find provider information, the participant shared that she thought this information should be made more accessible.

Just having the information available, online, I typed in the most obvious of questions and just couldn't, couldn't find anything … And I kind of get that it's not the kind of thing that clinics like, wants to advertise and stuff, but at the same time, it's kind of important that they do …. [P29, 21–25, Pacific Islander, home MA]

### 3.2 Demystifying Abortion

Throughout many accounts there was a sense that abortion had to be demystified, speaking to the stigma and resulting silence around abortion experiences. A variety of sources were consulted to do this, ranging from provider websites to personal blogs. In this way, OSI played a role in setting expectations and helping people feel prepared.

Many participants searched online because they did not know what an abortion involved or what it was like to have one; in many cases, this was how people first found out what happens. The following excerpt from a participant who used a provider website was a typical statement:

… [I] just read through everything, all the information that was on their site, and I think that's how I found out about what happens … [P01, 31–35, white British, home MA]

In multiple accounts, people sought to understand the bounds of what a ‘normal’ abortion was like. Information found online, particularly, around side effects, provided a point of reference that allowed them to feel reassured during their abortion and forewarned about potential issues.

Because I'd read all about it as well before, like this could happen, there should be this amount of blood. But because I read about it, I knew that was like my normal. [P08, 31–35, white British, home MA]

Expressing a desire to search for the worst possible outcomes, the same participant highlighted the opportunity that online spaces provide to find the extreme cases and to hear about a range of outcomes.

I also kind of like hearing the worst‐case scenario just so I can have in my head, like, what might happen. [P08, 31–35, white British, home MA]

Information found online played a direct role in how participants' expectations were set regarding their abortion. Some women shared that what they found aligned with their eventual experiences and as a result “… there wasn't anything that happened that was like a surprise.” [P01, 31–35, white British, home MA]. Other participants' expectations were not met, however. Reading information online caused one woman to expect the worst, and she expressed in visceral terms the feeling of fear around her abortion that was, in the end, was unfounded.

Like it was all very like, like intense, kind of compared to my actual experience, like, I really did think it was going to be a terrifying, horrific thing. [P18, 36–40, white Canadian, home MA]

Another woman shared that reading about others' experiences online made her believe that the process would be easy, particularly regarding pain and bleeding. This proved to be at odds with her own experience and she explained “… it was definitely worse than expected.” [P43, 16–20, Asian Afghani, home MA].

In addition to using OSI before their abortion, several participants who had a MA shared that they went online during the abortion itself to manage their anxiety and check if what was happening to them was normal. This included those who had previously experienced a MA, such as P10.

I also looked after I took the first pill. I was, “What's this actually done? Is there going to be any side effects?” [P10, 31–35, white British, home MA]

Upon finding out at a hospital scan that she had not yet passed the pregnancy, one participant turned to Google for specific, detailed information about what this would look like. This was information she had not previously been given, and she decided to search for this online.

And then I was Googling what it was going to look like. And on the internet, it said it was mainly grey with blood … [P45, 21–25, white British, hospital MA]

Reading accounts of others' abortion experiences was a common practice, cited by several participants as an integral aspect of preparing for their abortion. This type of information was often seen as distinct from that of ‘formal’ health information or abortion provider websites. Abortion stories predominantly came from ‘informal’ sources like blogs or forums—Reddit was mentioned in numerous accounts—but were occasionally found on provider websites. Participants described how they valued stories from real people in addition to more instructional accounts of what the abortion would be like, getting advice and learning how the abortion felt rather than simply what would happen.

It was just like telling one person's story about the process from finding out they are pregnant to like get an abortion and how the abortion actually felt. Yes, it was very helpful. [P24, 26–30, white British, home MA]

Several participants reflected on what they would have liked more of from OSI, and this frequently centered around a desire for moderated online resources that portrayed a range of real‐life abortion stories. One woman shared that she thought it would be beneficial for providers to direct people to existing resources, such as blogs and forums, for this type of information, and another explained that she felt provider websites were “missing that personal experience from someone else” [P28, 26–30, white Hungarian, home MA].

Speaking to the desire for moderated spaces where information could be curated by a trusted source, participants reflected on the challenges of seeking abortion information online. Although most did not mention difficulties when searching for information, one person reflected on the difficulty of differentiating between reliable information and anti‐abortion websites. It was also common to encounter very negative personal stories of abortion online. This was challenging and impactful for several participants.

Yeah, there was like some scary ones … that was quite a big hinderance and very big impact on my thoughts. Although reading was meant to help me quite a lot, there were one or two things that stuck in my mind, even now actually. [P29, 21–25, Pacific Islander, home MA]

Perhaps holding an awareness of the potential to come across such negative experiences, the same participant described how she actively sought “positive… abortion stories.” [P29].

### 3.3 Connection and Solidarity

Participants found connection and a sense of solidarity in online spaces; at times this was actively sought but in other cases it appeared incidental. Feeling connected to other people predominantly occurred through one‐way interactions, such as reading a blog or watching a video about another person's experience of abortion. While this practice could facilitate the process of learning about what happens through the abortion process, as described in the previous section, it also provided emotional support and a sense of shared experience. These aspects of engaging with abortion stories online cannot be completely detangled, however we present them as distinct motivations to explore what they can tell us about different needs during the experience of abortion. Participants made no reference to publicly sharing their own experiences online.

Finding others' abortion stories reassured people through the knowledge that they were not alone, and their experience was not exceptional.

It's sort of nice to know that, not nice, necessarily, but a lot of people are going through it, the same as you. [P08, 31–35, white British, home MA]

Where one participant had not had this experience, she explained:

… if I had the chance to read up about other women and what they experience and what they did and you know, that would have made me feel less alone with the whole experience … I think I would have liked to access a website or a folder or a handbook that I could read about other people's experiences …” [P28, 26–30, white Hungarian, home MA].

Although there was an awareness of the possibility of harassment in groups where people shared their own stories there was also a sense of community, and participants described feeling supported online. In the absence of assistance from her partner, friends, or family, one woman shared that Reddit was where she found most of her support. She described reaching out to strangers on the platform and the value she placed on the anonymity of the connection.

There are people on Reddit, you can reach out to them … they are a stranger but at the same time that helps. You just reach out and message them. It's all very informal. [P03, 21–25, British Bangladeshi, home MA]

The desire for an online support group was expressed by several people, to connect with others going through the same experience. One participant highlighted the difference between abortion and other health experiences, describing how anti‐abortion rhetoric has impacted the ability for these online spaces to be established safely.

There needs to be some kind of support group where you can talk to other women who are going through exactly what you're going through at exactly the same time. And I was so frustrated because in every other healthcare thing, there's Facebook groups about it … You can't get that in abortion because they just get filled up by all of the people who hate abortion and think it's a sin and murder. [P45, 21–25, white British, hospital MA]

### 3.4 Engaging With Pregnancy Content

The use of OSI to search for explicitly pregnancy‐related content arose in multiple participant accounts. In some cases, this involved pregnancy apps or websites to research symptoms while waiting for the abortion. Some participants additionally described engaging with online material in a way that explored their relationship with the pregnancy. One participant described the “curiosity” that had motivated her to search for specific details about the fetus, and the way this made her feel closer to the pregnancy.

And also just general pregnancy pages, like finding out whether my symptoms were normal. How big the fetus was at the time. And there was a point where I even checked when my actual due date would be. Just out of curiosity really … Yeah. I grew, I guess fond is the wrong word, but I grew quite attached to it, I guess. [P03, 21–25, British Bangladeshi, home MA]

Another participant used the Internet to find out what her child would look like. She expressed that this did not make the experience harder. Rather, it was part of her process to have a better understanding of “who” she was aborting.

I did go on one of those websites where you can put your photographs in and see what the child would look like. Yeah. But that didn't really make it much more difficult … Because I wanted to know who I'm aborting. [P10, 31–35, white British, home MA]

Participant accounts such as these suggest the nuanced and varied feelings that can exist within a person as they move through the abortion process. These feelings were brought up, intentionally or otherwise, through the use of OSI, as women explored and engaged with different types of pregnancy content.

## 4 Discussion

This article captures the varied and important role OSI plays in providing information, support, and a place for exploration throughout the abortion process. For some, there were challenges in the form of being unable to find the desired resources and encountering—or having concerns around encountering—incorrect or stigmatizing information.

The use of the internet is ubiquitous, and this was reflected in the way descriptions of OSI were integrated into participant accounts. Importantly, many described finding out how they could obtain an abortion using OSI: sourcing abortion services was a key and consistent reason for using OSI. Recent evaluations of online resources to support abortion access in parts of the United Kingdom and Ireland noted that although a large quantity of information is available online, there are challenges for users in identifying misinformation and evaluating information quality [20], with some resources not meeting the user's needs [37]. While studies such as these have identified challenges, some of which were echoed by people in this study, most participants found that OSI aided them in accessing services and appreciated the lack of judgment and supportive nature of these sources of information. This highlights the key role OSI plays in accessing abortion care, and is reflected in the body of research that focuses on the use of the internet to do so [21, 38, 39, 40].

OSI helped people to feel prepared. In a practical sense, this was often described as using OSI to learn about what happens during an abortion. Several participants who had a MA described searching online during their abortion to understand what was happening to them, highlighting the capacity of online resources to provide immediate support. This may be particularly beneficial for those having home‐managed abortions where there is an additional responsibility of care placed on the individual. OSI may also help to provide information that is considered “sensitive” and therefore at risk of being overlooked or omitted by providers [41, 42], such as what the pregnancy will look like when it passes.

The function of abortion stories was multiple across participant accounts, helping people feel both prepared and less alone in their experience. Stories were frequently not found on provider websites, but instead were read on blogs or forums where participants in this study gained insights from other people's lived experience.

Although many people in this study sought and desired to hear real abortion stories online, participants expressed wanting and needing different things from this practice. For some, hearing challenging, “horror stories” of abortion was a negative experience, whereas others specifically sought out the worst‐case scenario to feel prepared. Regarding their own experience, participants in this study spoke positively of using OSI where their own abortion aligned with what they had seen online. However, at times, the information they found did not match their experience, leading to either unnecessary fear or a false sense of being prepared. Taken together, this highlights the need to represent a diverse range of experiences when providing abortion information, giving people the opportunity to both find the kind of information they want in addition to seeing their own experiences represented. Previous research has identified the setting of expectations as an important aspect of abortion care [43, 44, 45, 46], however, few studies have explored the role of OSI in doing so. Our findings suggest that online information, particularly real stories of abortion, could be well placed to support this important aspect of quality abortion care.

Through engaging with content that highlighted others' experiences, people's notions of abortion as exceptional and isolating were challenged. As stigma can limit open discussion about abortion, there has been increasing interest from researchers and pro‐abortion rights activists in the potential for storytelling and experiential information to normalize abortion and provide support [11, 47, 48, 49, 50]. A number of mediums have been explored to share abortion stories online, including animation [51], story submission platforms [52], and digital storytelling websites [53]. However, work in this area has often focused on reducing stigma in the general public, and there has been limited evaluation of how storytelling could provide this kind of support for people who are seeking or having abortions. In an analysis of abortion representation online and in popular media, Baird and Millar call for caution around assumptions that abortion stories are inherently helpful in countering stigma and normalizing the experience [11]. The authors call for representation that goes beyond “one dimensional” portrayals, for example, solely unapologetic or solely regretful accounts, that do not fully move beyond the prevailing narratives of abortion as a negative and uncomplex experience. Research that explored how people share their abortion stories online highlighted the work people undertake to tell their stories and either resist or conform with such narratives [54], and the tensions that can impact people's ability to share and make sense of their own stories [55]. These perspectives are important when considering the potential supportive role of real accounts for people having abortions.

While encountering other's stories was described as making people feel less alone, only one participant described engaging in a two‐way interaction online, where she privately messaged other users on Reddit for support around the abortion experience. This kind of interaction has been captured in previous studies, where online spaces were used to find community around abortion, communicate back and forth with others, and address perceived gaps in in‐person support [17, 26, 56]. Participants in this study did not mention sharing their own abortion stories on a public platform, a practice that has been described in other research [17, 56]. People did, however, express a desire for safe, moderated online spaces that could facilitate two‐way communication and connection with others going through the same experience, a finding shared by previous work on abortion stigma and support [57]. These experiences also speak to work that has identified emotional and interpersonal support as an area that people having abortions feel is lacking in current clinical care in Britain [6]. Online social support groups for people who have experienced miscarriage has been explored with some positive findings [58]. However, further research is required to better understand the opportunities and challenges this presents for those with experience of abortion.

Using OSI to engage with pregnancy content was not a dominant theme across our interviews. However, when discussion around this topic arose it appeared to be distinct from other motivations for seeking information online, arising from a place of curiosity. It has been acknowledged that the internet is a place where complex and contradictory feelings can be explored in the context of abortion, offering a space where topics that may otherwise have remained undisclosed and unseen are made visible [26, 59]. Online spaces allow information to be accessed anonymously and may facilitate the process of making sense of a nuanced and potentially challenging experience. In recognizing the curiosity with which some participants approached engaging with their pregnancy, we can acknowledge the role of this practice in making sense of the abortion experience. Further research to better understand how people seeking or having abortions interact with pregnancy content and information would be valuable.

While some people encountered stigmatizing content when using OSI, or were aware of the possibility of doing so, the internet was also a site where people resisted or rejected stigma, an idea explored in recent research on online spaces and social support for abortion [17]. For our participants, this negotiation of stigma occurred when people actively sought out positive abortion stories to avoid the anticipated negative portrayals of abortion and the feelings of solidarity experienced when people learned that their experience was not exceptional. In this way, OSI can be places where abortion is normalized and contextualized. Proactive signposting to supportive resources, be it by healthcare professionals, charities, or social networks, has been suggested as a way to mitigate encountering stigmatizing content online [17], and our findings support this.

Our findings also highlight people's desire for experiential abortion information and opportunities to feel connected to a wider community of people with similar experiences, as well the room for nuanced exploration afforded by online spaces. Many participants had experienced a previous abortion(s), suggesting the importance of OSI even when an individual has personal knowledge of the abortion process. However, the varied views of our participants made it clear that people want and need different things from the OSI they consult, raising questions around what OSI for abortion should look like to support these preferences. How can diverse, representative stories be shared and found in non‐stigmatizing, supportive online spaces, and whom should these be provided by? OSI that deliver this have the potential to not only to provide knowledge and support, but also to normalize abortion and challenge stigma, particularly for those seeking or having abortions.

While exploring the potential for online resources to support people further, recognition is needed of the potential for this approach to widen existing inequalities. The need for digital literacy is a central concern when investigating new healthcare interventions [60], and lower digital literacy is associated with sociodemographic factors, such as income level, education level, immigration status, ethnicity and disability [61, 62]. Ensuring that any developments in abortion care are adapted and suitable for all who need them is crucial to enable already underserved populations to benefit from them.

Future research should focus on developing and evaluating OSI and digital interventions that meet people's needs for informational—instructional and experiential—and support, and integrating these into abortion services. While what people want from OSI varies, particular attention should be paid to the value of real abortion stories and opportunities to find connection around the abortion experience.

### 4.1 Strengths and Limitations

The sample was recruited from both NHS and independent provider sites and included a diverse range of ages and ethnicities, abortions carried out in all British countries, and a representative abortion method split [4]. These factors together suggest that a wide range of perspectives and experiences will have been included. Unlike many other studies that engage with the use of OSI for abortion, this project was not restricted to exploring one OSI or platform and instead considered the use of the internet for information more broadly. As such, this research was well placed to capture a wider range of needs and motivations. As far as we are aware, this is the first study to qualitatively explore how people having abortions across Britain use OSI.

Considering the limitations of this research, it is important to note that information on a number of characteristics was not gathered—for example, disability, gender identity, sexual orientation, or a measure of socioeconomic status—and, therefore, it is not possible to ascertain who is excluded from the sample in relation to these characteristics. Furthermore, due to the study inclusion criteria, the experiences of those under 16 years old and people who could not speak English, Arabic, Polish, or Welsh were not able to participate. Importantly, therefore, this research is not representative of the wider population. Only a small number of participants had procedural abortions, and, although this is reflective of the wider population, there may be experiences around OSI that are specific to those having procedural abortions that are not captured, and it would be valuable to explore these. While interviewing people with very recent experience of abortion was useful for their recollection, the immediacy with which some interviews were conducted may have meant that the longer‐term use of OSI was not captured. Finally, the experiences and motivations detailed in this paper are from a context where abortion was obtained through a legal route, with remote or in‐person clinical support. The importance of the internet in regions where there are restrictive abortion laws has been highlighted in previous work [63]; while there may be similarities, the role OSI play in settings with different legal frameworks and levels of clinical involvement may vary.

### 4.2 Conclusions

Our study provides new insights into the role played by OSI for people having abortions. Our analysis of people's accounts generated four motivations for using OSI: (1) accessing services; (2) demystifying abortion; (3) connection and solidarity; and (4) engaging with pregnancy content.

While people encountered challenges when navigating online spaces, OSI were an important resource for information, connection, and exploration, as well as a space where stigma could be negotiated. The potential for OSI to fulfill these roles, however, has not been fully explored. The findings of this study suggest that OSI could be an important resource through which to improve people's experience of abortion in Britain: a site of intervention to ensure people have the information and support they need throughout the abortion process. The motivations for OSI use, such as those detailed in this article, should guide the focus of policy and practice developments when creating online resources for those having abortions.

## Author Contributions

R.Ma. and R.S. conducted the data analysis, and R.Ma. wrote the manuscript. R.S. conceptualized the research question for this paper, and R.S. and R.Ma. developed and refined it over the course of the research. K.W. and R.S.F. designed and led the entire SACHA study. K.W. and P.A.L. led the qualitative work package. R.S., M.L., R.Me., N.S., P.A.L., S.C., M.P., K.W., and R.S.F. contributed to the study design and protocol. R.Me. and M.L. coordinated the ethical approvals. P.A.L. and S.C. facilitated clinical fieldwork. M.L. organized participant recruitment and training for recruiting sites. M.L., N.S., and R.S. conducted participant recruitment. M.L., R.S., R.Me., N.S., and K.W. conducted the interviews. All authors contributed comments to the final manuscript. Investigators of the SACHA Study provided feedback on all stages of the study.

## Ethics Statement

Ethical approvals were obtained from the Research Ethics Committees of the British Pregnancy Advisory Service (reference 2021/02/WEL), the London School of Hygiene & Tropical Medicine (reference 22,761), *LSHTM MSc Research Ethics Committee (Ref: 29518)*, and the NHS (reference 21/LO/0236).

## Patient and Public Involvement (PPI)

The aim of this research was to ensure that patients' voices informed abortion policy and practice. PPI members of the Advisory Group provided input on the interview guide and Participant Information Sheet.

## Conflicts of Interest

The authors declare no conflicts of interest.

## Data Availability

The data are subject to ethical approvals and so cannot be shared. More information about the data can be sought from the corresponding author. Thanks are due to the SACHA Study team^†^ as a whole for contributing ideas that were instrumental to this research: Annette Aronsson, Paula Baraitser, Caroline Free, Louise Keogh, Clare Murphy, Wendy Norman, Jennifer Reiter, Jill Shawe, Sally Sheldon, and Geoffrey Wong. We would also like to thank our Advisory Group for supporting the development of this study, and the stakeholders who helped us contextualize findings and prioritize areas for improvement in abortion care in Britain.
